# Reducing the Intake of Sodium in Community Settings: Evaluation of Year One Activities in the Sodium Reduction in Communities Program, Arkansas, 2016–2017

**DOI:** 10.5888/pcd15.180310

**Published:** 2018-12-20

**Authors:** Christopher R. Long, Brett Rowland, Krista Langston, Bonnie Faitak, Karra Sparks, Victoria Rowe, Pearl A. McElfish

**Affiliations:** 1 College of Medicine, University of Arkansas for Medical Sciences Northwest, Fayetteville, Arkansas; 2 Office of Community Health and Research, University of Arkansas for Medical Sciences Northwest, Fayetteville, Arkansas

## Abstract

**Purpose and Objectives:**

The Centers for Disease Control and Prevention’s Sodium Reduction in Communities Program (SRCP) aims to reduce dietary sodium intake through policy, systems, and environmental approaches. The objective of this study was to evaluate and document the progress of the first year of a 5-year SRCP project in northwest Arkansas.

**Intervention Approach:**

In collaboration with 30 partner schools and 5 partner community meals programs, we sought to reduce dietary sodium intake through increased implementation of 1) food service guidelines, 2) procurement practices, 3) food preparation practices, and 4) environmental strategies.

**Evaluation Methods:**

We collected daily menus, information on nutritional content of meals, and procurement records and counted the number of people served in partnering schools and community meals programs. We used a pretest–posttest quantitative evaluation design to analyze changes in the sodium content of meals from baseline to Year 1 follow-up.

**Results:**

From baseline to Year 1 follow-up, participating schools lowered the mean sodium content served per lunch diner from 1,103 mg to 980 mg (−11.2%). The schools also reduced the mean sodium content of entrées offered (ie, entrées listed on the menu) from 674 mg to 625 mg (−7.3%) and entrées served from 615 mg to 589 mg (−4.2%). From baseline to follow-up, participating community meals programs reduced the mean sodium content of meals offered (ie, meals listed on the menu) from 1,710 mg to 1,053 mg (−38.4%). The community meals programs reduced the mean sodium content of meals served from 1,509 mg to 1,258 mg (−16.6%).

**Implications for Public Health:**

In both venues, our evaluation findings showed reductions in sodium served during the 1-year evaluation period. These results highlight the potential effectiveness of sodium reduction interventions focused on food service guidelines, procurement practices, food preparation practices, and environmental strategies for schools and community meals programs.

## Introduction

The *2015–2020 Dietary Guidelines for Americans* recommends that daily dietary sodium intake not exceed 2,300 mg for people aged 14 years or older (1). However, people in the United States consume more sodium than is recommended (2–4). Among Americans aged 2 years or older in 2013–2014, males consumed a mean of 3,915 mg of sodium per day, and females consumed 2,920 mg (5).

Approximately 25% to 30% of US adults have hypertension (6,7). Hypertension is strongly associated with risk for cardiovascular disease (8), the leading cause of death in the US population (6). Consensus on dietary sodium intake is that sustained excessive sodium intake is associated with hypertension and increased risk for cardiovascular disease and that reducing excessive sodium intake has a direct effect of lowering blood pressure (9–14). Across a range of approaches, health impact assessment models consistently predict sizeable health benefits of reduced sodium intake (15). An analysis published in 2017 indicated that a 10% reduction in sodium intake worldwide over 10 years would avoid 5.8 million disability-adjusted life years (16).

The Centers for Disease Control and Prevention (CDC) implemented the Sodium Reduction in Communities Program (SRCP) to achieve the benefits of reduced dietary sodium intake across large populations in the United States by reducing sodium intake to recommended levels (17,18). Program awardees are charged with increasing access to healthy, lower-sodium foods in venues that serve food to relatively large numbers of community members (19). Program activities focus on increasing the number of lower-sodium foods offered rather than restricting food choices. Program venues include correctional facilities, early childhood education centers, institutions of higher learning, hospitals, worksites, and others (18). Each awardee is required to evaluate the effectiveness of the strategies in its targeted venues (19).

## Purpose and Objectives

In 2016, the University of Arkansas for Medical Sciences (UAMS) received a 5-year SRCP award to implement sodium reduction strategies in northwest Arkansas in public school cafeterias and in community meals programs (programs that offer free meals to low-income patrons). UAMS and local stakeholders selected these venues because they serve populations in northwest Arkansas at elevated risk for hypertension, namely Pacific Islander, low-income, and food-insecure populations (6,7,20). This project presented a unique opportunity to evaluate the effects of the simultaneous implementation of multiple sodium reduction strategies in 2 venues. The objective of our study was to describe the strategies, intervention, and outcomes during Year 1 of UAMS’s SRCP project.

Before applying for an SRCP award, UAMS assembled an internal team of researchers, a registered dietician, policy experts, and staff with experience in implementing health-related interventions in food system venues. UAMS also engaged key stakeholders in northwest Arkansas. These stakeholders represented local community meals programs, school districts, large employers, vendors, community groups, and a center for culinary arts. Stakeholders engaged in quarterly group meetings and monthly one-on-one meetings with UAMS. These meetings focused on discussions about their interest in and capacity to support an SRCP project in various potential venues. UAMS and stakeholders agreed that school districts and community meals programs should be selected as venues.

### School districts

The public school districts in northwest Arkansas serve food daily to more than 100,000 students and staff (21). Several school districts were particularly enthusiastic about participating in SRCP because of planned changes to the US Department of Agriculture’s (USDA’s) school lunch policy. The USDA’s proposed standards required schools participating in the National School Lunch Program to comply with reduced sodium standards. For example, standards for high school cafeterias reduced the allowable amount of sodium in lunches from an average of 1,588 mg to 1,420 mg or less in 2014 and — if implemented as scheduled — will further reduce the allowable amount of sodium to 740 mg or less in 2022 (22).

UAMS selected the public school district in Springdale, Arkansas, as the first school district partner for project implementation because of its socioeconomic and health-related challenges. In 2017, Springdale school district cafeterias served more than 24,000 students and staff daily (23). Among Springdale’s more than 20,000 students, the prevalence of overweight/obesity was 43% in school year 2016–2017 (24). Many of Springdale’s students came from low-income households and were Pacific Islanders; both groups are associated with an increased risk for hypertension (6,7). Approximately 71% received free or reduced-price lunch (25), higher than the prevalence observed in the United States (51.8%) and Arkansas (62.3%) (26). Approximately 13% of the school district’s students were Marshallese (Pacific Islander) (27).

### Community meals programs

In 2016, northwest Arkansas community meals programs served approximately 4,000 people daily. These community meals programs included free community meals served on site (eg, in soup kitchens) and weekend food bags for children to supplement their weekend meals. These programs were selected because many of their patrons have health challenges associated with food insecurity, homelessness, poverty, and unemployment. Food insecurity and low income are associated with increased risk for hypertension (6,20). Five community meals programs were selected as Year 1 partners for project implementation. These programs were selected on the basis of the following 4 criteria: 1) their reach (ie, the programs’ self-reported collective reach was ~3,000 meals per day), 2) their diversity of approach (eg, 3 programs served meals on site and 2 programs provided weekend food bags for children), 3) their diversity of location (ie, throughout northwest Arkansas), and 4) their willingness to participate.

### Intervention components

Intervention components at each venue were based on increased implementation of 4 broad strategies recommended by SRCP: 1) food service guidelines that discuss sodium, 2) procurement practices to reduce sodium content in foods and ingredients purchased, 3) food preparation practices to reduce sodium content of menu items and meals, and 4) environmental strategies that encourage reductions in dietary sodium intake. The effectiveness of these 4 components was evaluated at each venue according to the following 4 evaluation questions, common to all SRCP projects:

1. How and to what extent have sodium reduction interventions been implemented in specific venues and entities?
2. How and to what extent has the food environment changed since the implementation of sodium reduction interventions, specifically addressing availability of lower-sodium food products?
3. To what extent have lower-sodium food products been purchased or selected by either consumers or larger service providers?
4. What promising and innovative sodium reduction strategies have been found effective that could be replicated by similar communities (28)?

## Intervention Approach

Upon notification that UAMS’s application was successful, UAMS convened a food policy committee for each venue (Figure). For the school district, the food policy committee consisted of child nutrition administrators, and they scheduled monthly meetings; however, they met 7 times during Year 1. For the community meals programs, the committee consisted of staff responsible for administration, procurement, operations, and food preparation for each program. The community meals committee initially met monthly but then changed to bimonthly after feedback from committee members; they met 10 times during Year 1.

**Figure:**
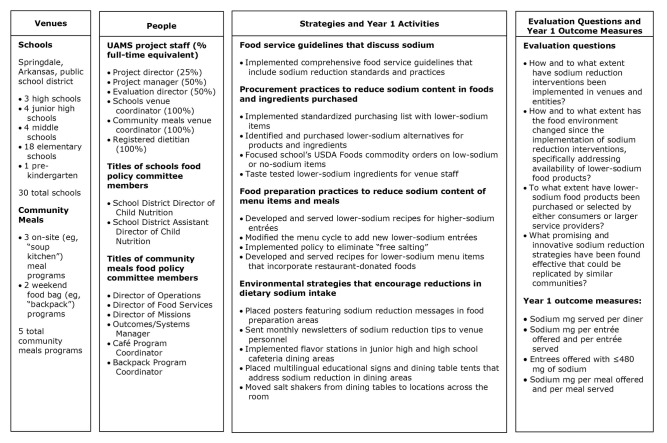
Overview of implementation of the Sodium Reduction in Communities Program, Arkansas, 2016–2017. Abbreviations: UAMS, University of Arkansas for Medical Sciences.

To prepare for each committee meeting, SRCP staff researched potential implementation strategies, prepared materials, and developed examples of how each venue could implement each of the 4 SRCP strategies. During committee meetings, project staff presented and discussed this information. For example, during an initial meeting with the school food policy committee, project staff proposed options for environmental strategy implementations (eg, hanging posters featuring sodium reduction messages in food preparation areas of school cafeterias, rearranging dipping sauces on the lunch line to make lower sodium options more accessible), and the committee selected the options they wanted to implement. Topics at subsequent food policy meetings included targeting and modifying high-sodium recipes to reduce sodium and identifying educational materials most suitable for each location. All implementation of intervention strategies in both venues resulted from decisions made in committee meetings. In addition, food policy committees could choose to reject, partially implement, or delay activities until Year 2 or later (Table 1).

**Table 1:** Rejected, Partially Implemented, or Delayed Intervention Activities Presented to the Food Policy Committees at Schools and Community Meals Programs Participating in the Sodium Reduction in Communities Program, Northwest Arkansas, 2016–2017

Intervention Strategies and Activities	Food Policy Committee Decision^a^	Reason for Decision
**Schools**		
**Procurement practices to reduce sodium content**		
Form a purchasing cooperative with neighboring school districts to negotiate favorable prices for lower-sodium products and ingredients	Reject	Districts were served by different vendors and had very different menus and student populations
Remove high-sodium items from the menu, including pizza and cookies	Reject	District personnel indicated that these items were popular with students
**Food preparation practices to reduce sodium content of menu items and meals**		
Implement recipe modifications developed by students at local center for culinary arts	Partially implement	Many proposed recipes were impractical because of expense and number of ingredients and use of uncommon or noncommodity ingredients
Increase use of fresh ingredients (eg, herbs, vegetables) to add flavor in place of salt	Delay	Food preparation staff lacked time to devote to preparing additional fresh ingredients; insufficient number of staff with sufficient knife skills
**Environmental strategies that encourage reductions in dietary sodium intake**		
Place posters featuring sodium reduction messages in student dining areas of cafeterias	Delay	District personnel wanted to delay implementation to generate student enthusiasm by placing posters at the beginning of a new school year
Re-order list of menu items on digital menus to highlight lower-sodium items	Delay	Staff lacked time and knowledge to reprogram digital signage
Rearrange drinks in coolers to promote lower-sodium options	Delay	Some coolers (eg, those with fixed shelving) could not be reconfigured to highlight lower-sodium options
Purchase and implement upgraded displays (eg, fruit baskets) to promote lower-sodium options	Delay	The 2015–2016 equipment purchasing cycle had ended
**Community Meals Programs**		
**Procurement practices to reduce sodium content**		
Reduce the amount of high-sodium–donated restaurant food served	Reject	Community meals programs expressed concern that they could not afford to purchase enough lower-sodium food to replace high-sodium–donated restaurant food
Replace canned vegetables at 1 program with lower-sodium frozen vegetables	Reject	Community meals program indicated it lacked sufficient freezer space (freezer space was filled with donated restaurant food)
Remove donuts from meals at 1 program	Reject	Community meals program indicated that donuts were popular with diners
Implement new lower-sodium recipes	Partially implement	Community meals programs expressed concern about the expense and difficulty of acquiring several lower-sodium ingredients from vendors and stores
**Food preparation practices to reduce sodium content of menu items and meals**		
Increase use of fresh ingredients (eg, herbs, vegetables) to add flavor in lieu of salt	Delay	Food preparation staff lacked time to devote to preparing additional fresh ingredients; staff lacked consistent access to low-cost fresh ingredients
Replace prepackaged salad dressings with lower-sodium dressing made on site	Reject	One community meals program indicated that salad dressing was often received as a donation, so they did not want to spend budget to make their own
**Environmental strategies that encourage reductions in dietary sodium intake**		
Implement flavor stations in dining areas to replace salt shakers	Reject	Community meals programs expressed concerns about food safety and disruption of the flow of diners through the serving area while using flavor stations

a “Reject” indicates that the food policy committee declined to implement the activity. “Partially implement” indicates that the food policy committee implemented some components of the activity but not all. “Delay” indicates that the food policy committee decided to delay implementation of the activity until project Year 2 or later.

During food policy committee meetings, project staff discussed implementation challenges and successes, solicited committee’s feedback on implementation progress, and collaborated with committee members to identify potential improvements. Project staff aimed to minimize the time and effort required from committee members by limiting meetings to approximately 1 hour.

### Intervention activities in the school district

At the project’s beginning, UAMS’s registered dietitian and other UAMS staff engaged school district personnel in discussions to augment existing school district nutrition policies to include sodium-focused annual health and nutrition training to all cafeteria staff. This intervention activity provided a policy foundation for other intervention activities. The school district’s Child Nutrition Department centrally managed the district’s child nutrition policies, procurement, and food preparation practices, so any changes implemented by the Child Nutrition Department would affect almost all food served in the district’s 29 cafeterias. One of the district’s 30 schools, a stand-alone prekindergarten facility, did not have on-site lunch preparation and was unable to participate in Year 1 activities, although students and staff did have access to lunch prepared at participating schools. 

Throughout the year, UAMS staff engaged school district personnel to implement procurement practices to reduce sodium content in foods and ingredients purchased by the school district. The school district personnel involved in implementing these practices included the Child Nutrition Director, Child Nutrition cafeteria managers, and food service staff. Procurement practices to be implemented included 1) developing a standardized purchasing list to increase ordering of lower-sodium items, 2) focusing the school district’s USDA Foods commodity orders on low-sodium and no-sodium items, and 3) identifying and purchasing lower-sodium alternatives for products and ingredients. To encourage procurement of lower-sodium foods and ingredients, the UAMS registered dietitian and a registered nutrition and dietetic technician taste-tested lower-sodium recipes with district personnel.

At the same time, UAMS staff worked with school district personnel to implement food preparation practices to reduce sodium content of menu items and meals. Food preparation practices included 1) collaborating with students from a local center for culinary arts to develop lower-sodium recipes for higher-sodium entrées identified by school district personnel and 2) modifying the menu cycle to add new lower-sodium entrées. Entrées were classified by school district personnel as food that met the USDA’s definition of “meat/meat alternate” and was served as a main dish (29). UAMS and school district personnel aimed to reduce sodium content of all entrées on the lunch menu to 480 mg or less by Year 5 and adopted the USDA’s Smart Snacks in School sodium guideline for entrées as a target (30). In addition, UAMS staff worked with school district personnel to implement environmental strategies that encourage reductions in dietary sodium in school lunches. Environmental strategies included 1) an educational campaign that placed posters featuring sodium reduction messages in dining areas of school cafeterias, 2) an educational campaign that placed posters featuring sodium reduction messages in food preparation areas of school cafeterias, 3) a monthly newsletter of sodium reduction tips sent by UAMS staff to venue personnel, and 4) implementation of flavor stations in junior high school and high school cafeterias, presenting diners with the choice to add a range of low-sodium and no-sodium seasonings to their meals.

### Intervention activities in community meals programs

In the community meals programs, intervention activities were similar to activities in the school district. However, in contrast to the centralized organizational structure of the school district, each community meals program had its own organizational structure, policy environment, and operating procedures. To encourage sharing of knowledge among the community meals programs and to facilitate communication between the UAMS team and community meals program staff, representatives from all 5 programs were invited to semi-annual peer learning-exchange meetings hosted at UAMS. These meetings included lower-sodium food preparation demonstrations, lower-sodium product taste-testing (eg, lower-sodium versions of ranch dressings, salsas, and marinara sauces), and data sharing between UAMS staff and community meals program staff.

At the project’s beginning, UAMS staff engaged community meals program staff in discussions to either establish nutrition policies or augment existing policies to incorporate food service guidelines that discuss sodium. At each program, the UAMS registered dietitian and other UAMS staff collaborated with community meals program personnel to develop a work plan and comprehensive food service guidelines that include sodium reduction. As with the school district, this intervention activity was intended to provide a unifying rationale for the other intervention activities in the community meals programs.

Throughout the year, UAMS staff engaged community meals program staff to implement procurement practices to reduce sodium content in foods and ingredients. The UAMS registered dietitian and other UAMS staff encouraged personnel at each program to create a standardized food purchasing list, and the UAMS registered dietitian and registered nutrition and dietetic technician identified the most commonly purchased ingredients and presented and taste-tested lower-sodium alternatives with community meals program staff.

UAMS staff also worked with the food service staff (sometimes including food service volunteers) at each community meals program to implement food preparation practices to reduce sodium content of menu items and meals. For example, a policy to eliminate “free salting” (ie, adding unmeasured quantities of salt at the end of meal preparation) was encouraged. Also, after UAMS staff identified that restaurant-donated foods were a primary contributor to the highest-sodium meals served at the venue, the UAMS registered dietitian worked with community meals program staff to develop recipes for lower-sodium menu items that incorporated restaurant-donated foods (eg, lowering sodium by adding cooked dry black beans and rice to restaurant-donated “chicken burrito bowls”). In addition, UAMS staff worked with community meals program staff to implement environmental strategies that encouraged reductions in dietary sodium in the meals served. Environmental strategies to be implemented included 1) consultation with venue staff to create and place multilingual (ie, English, Marshallese, and Spanish) educational signs and table tents that addressed sodium reduction and health concerns common to patrons and 2) moving salt shakers from the dining tables to a location across the dining room.

## Methods

SRCP requires annual evaluation of project progress. To meet this requirement, we used a pretest–posttest quantitative evaluation design at each venue. We selected this design because it facilitated monitoring progress toward project objectives (eg, reduction in community members’ sodium intake) at each venue, and it provided standardized quantitative indicators that 1) can be collected repeatedly across the life of the project, 2) were responsive to each evaluation question, and 3) can be aggregated by CDC across projects in its overall evaluation of SRCP. In addition, this approach saved costs by leveraging nutrient data, daily diner counts, procurement records, and daily food production records that the schools were required to collect as part of other regulatory obligations.

We collected data at each venue immediately before intervention implementation and again 10 or 11 months later, minimizing variability due to seasonal factors (eg, seasonal changes in availability of fresh fruits and vegetables). In the school district, we collected baseline data during 2 consecutive weeks of meals in December 2016 and follow-up data during 2 consecutive weeks of meals in October 2017. In the community meals program, we collected baseline data during 4 consecutive weeks of meals in January 2017 and follow-up data during 2 consecutive weeks of meals in October 2017.

We included in evaluation data collection all schools or community meals programs that implemented sodium reduction interventions. The data sources for the schools venue evaluation included annual procurement records, daily food production records, daily counts of people served per school, menu item nutrient reports, and the UAMS team’s implementation records. Food production records, counts of people served, and menu item nutrient reports were generated for each school by school district staff using PrimeroEdge school nutrition software (Cybersoft Technologies, Inc) and shared with the UAMS team. Daily sodium information for each menu item at baseline and follow-up was included as part of the menu item nutrient report and was based on USDA’s Child Nutrition database (31).

The data sources for the community meals venue evaluation included the UAMS team’s implementation records and each program’s weekly or monthly procurement records, daily menus, and daily counts of people served. In addition, the UAMS registered dietitian and other UAMS staff visited each program each day it was open during the data collection period, observing and documenting how food was prepared by community meals program staff. The documentation process included recording amounts of each ingredient used (weight or volume, depending on the ingredient and method of preparation), names of all food products used, pictures of food product labels, and menu item serving sizes. The UAMS registered dietitian calculated the daily sodium value for each menu item at baseline and follow-up by entering ingredient and serving size data into Nutritionist Pro software (Axxya Systems, LLC), which hosts a database of nutritional information for more than 80,000 foods.

For the schools venue, we evaluated point-of-service and sodium data from 193,232 diners served during 12 days at 28 schools during baseline data collection. During follow-up data collection, we evaluated point-of-service and sodium data from 173,087 diners served during 10 days at 29 schools. (We excluded 1 school from baseline calculations because of differences in menus, purchasing, and food preparation compared with other cafeterias in the district; at follow-up, the school had standardized its menus to match those of the other schools in the district and was included in follow-up calculations. We excluded the standalone pre-kindergarten site from both baseline and follow-up calculations because it did not have on-site lunch preparation.)

For the community meals venue, we evaluated point-of-service and sodium data from 13,319 meals served to diners during 12 days at all 5 programs during baseline data collection. During follow-up data collection, we evaluated point-of-service and sodium data from 10,136 meals served during 6 days.

We did not conduct power calculations because the evaluation 1) focused on descriptive analyses for outcomes and 2) sampled the entire population of participating entities in each venue. Statistical analyses were conducted in SPSS Statistics version 25 (IBM Corp) and Microsoft Excel version 15.0 (Microsoft Corp). Missing data were minimal, and we did not impute missing values. In the schools venue, data from only 2 (0.3%) of the 626 lunch services across the included cafeterias during the data collection periods were not recorded by cafeteria staff. In the community meals venue, no data were missing.

For each venue, we prepared data sets by aggregating all data from each entity without any weighting, allowing calculation of venue-level totals for number of diners served, mg sodium served, number of entrées offered, and other measures. For categorical or count variables, we tabulated venue-level counts and percentages. For continuous variables for which sodium mg was the unit of measure, we tabulated results as venue-level means. For example, in each venue, we calculated mean sodium mg served per diner by dividing the total sodium mg served across all participating entities during the data collection period by the number of diners served across all participating entities during the data collection period.

The evaluation was ruled exempt by UAMS’s institutional review board.

## Results

### Schools venue

Approximately 24,000 diners (~20,000 students and ~4,000 staff members or visitors) were exposed to the sodium reduction intervention in the schools venue daily during the school year. In general, 29 of 30 schools (96.7%) implemented the sodium reduction interventions (Table 2). Across the schools venue, the amount of sodium served per lunch diner during the evaluation period decreased 11.2%, from 1,103 mg at baseline to 980 mg at follow-up (Table 3). The schools also reduced the mean sodium content of entrées offered (ie, entrées listed on the menu) from 674 mg to 625 mg (−7.3%) and entrées served from 615 mg to 589 mg (−4.2%).

**Table 2:** Sodium Reduction Intervention Activities Implemented by Schools and Community Meals Programs Participating in the Sodium Reduction in Communities Program, Northwest Arkansas, 2016–2017

Intervention Strategies and Activities	No. (%) at Follow-Up^a^
**Schools (n = 30)**	
**Food service guidelines that discuss sodium**	
Implemented comprehensive food service guidelines that include sodium reduction standards and practices	29 (96.7)
**Procurement practices to reduce sodium content**	
Implemented standardized purchasing lists with lower-sodium items	29 (96.7)
Focused USDA Foods commodity orders on low-sodium or no-sodium items	29 (96.7)
Identified and purchased lower-sodium alternatives for products and ingredients	29 (96.7)
Participated in taste-tests of lower sodium ingredients for program staff	29 (96.7)
**Food preparation practices to reduce sodium content of menu items and meals**	
Developed and served lower sodium recipes for higher sodium entrées	29 (96.7)
Modified the menu cycle to add new lower sodium entrées	29 (96.7)
**Environmental strategies that encourage reductions in dietary sodium intake**	
Placed posters featuring sodium reduction messages in food preparation areas	29 (96.7)
Received monthly newsletters of sodium reduction tips sent by UAMS staff	29 (96.7)
Implemented flavor stations in junior high and high school cafeterias	7 (23.3)
**Community Meals Programs (n = 5)**	
**Food service guidelines that discuss sodium**	
Implemented comprehensive food service guidelines that include sodium reduction standards and practices	3 (60.0)
**Procurement practices to reduce sodium content**	
Implemented standardized purchasing lists with lower sodium items	2 (40.0)
Participated in taste-tests of lower sodium ingredients for program staff	4 (80.0)
**Food preparation practices to reduce sodium content of menu items and meals**	
Implemented policy to eliminate “free salting”	3 (60.0)
Developed and served recipes for lower sodium menu items that incorporate restaurant-donated foods	3 (60.0)
**Environmental strategies that encourage reductions in dietary sodium intake**	
Placed posters featuring sodium reduction messages in food preparation areas	3 (60.0)
Placed multilingual educational signs and dining table tents that address sodium reduction in dining areas	3 (60.0)
Received monthly newsletters of sodium reduction tips sent by UAMS staff	5 (100.0)
Moved salt shakers away from dining tables to locations across the room	3 (60.0)

Abbreviations: USDA, US Department of Agriculture; UAMS, University of Arkansas for Medical Sciences.

a Data were collected at each venue immediately before intervention implementation and again 10 or 11 months later. In the school district, we collected baseline data during 2 consecutive weeks of meals in December 2016 and follow-up data during 2 consecutive weeks of meals in October 2017. In the community meals program, we collected baseline data during 4 consecutive weeks of meals in January 2017 and follow-up data during 2 consecutive weeks of meals in October 2017. At baseline, none of the activities had been implemented at any of the venues.

**Table 3:** Baseline and 1-Year Follow-Up Outcome Measures for Sodium Reduction Interventions at Schools and Community Meals Programs Participating in the Sodium Reduction in Communities Program, Northwest Arkansas, 2016–2017

Outcomes	Baseline	Follow-Up	Percentage Change
**Schools (n = 30)^a^**			
Sodium per entrée offered, mg	674	625	−7.3
Sodium per entrée served, mg	615	589	−4.2
Entrées offered with ≤480 mg of sodium, no. (%)	26 (24.3)	38 (32.8)	+46.2
Sodium served per lunch diner, mg	1,103	980	−11.2
**Community meals programs (n = 5)**			
Sodium per meal offered, mg	1,710	1,053	−38.4
Sodium per meal served, mg	1,509	1,258	−16.6
Sodium served per diner, mg	1,509	1,258	−16.6

a Calculations at baseline and follow-up are based on data from 28 and 29 schools, respectively. One school was excluded at baseline because of differences in menus, purchasing, and food preparation compared with other cafeterias in the district; at follow-up, the school had standardized its menus to match those of the other schools in the district and was included in calculations. A stand-alone prekindergarten site was excluded from both baseline and follow-up calculations because it did not have on-site lunch preparation.

The recipes of 7 (2.5%) of the schools’ 277 lunch menu items were modified to reduce sodium content. For example, by using no-salt-added tortilla chips in place of regular tortilla chips, the sodium content of the taco salad entrée was reduced from 818 mg at baseline to 543 mg at follow-up, and the sodium content of the cheesy nachos entrée was reduced from 806 mg at baseline to 609 mg at follow-up. Twelve (4.3%) lunch menu items were modified through ingredient or product substitution to reduce sodium content. For example, by replacing breaded pork patties with pork patties made with a whole-grain breading that was lower in sodium, the schools reduced the sodium content of their pork sandwiches from 603 mg at baseline to 203 mg at follow-up.

### Community meals venue

Approximately 3,100 unique diners per day were exposed to the sodium reduction intervention in the community meals venue during the year. Adoption of sodium reduction intervention activities varied among sites; only 2 programs implemented standardized purchasing lists with lower sodium items, but all 5 programs received newsletters of sodium reduction tips sent by UAMS (Table 2).

The amount of sodium served per diner during the evaluation period decreased 16.6%, from 1,509 mg to 1,258 mg (Table 3). From baseline to follow-up, participating community meals programs reduced the mean sodium content of meals offered (ie, meals listed on the menu) from 1,710 mg to 1,053 mg (−38.4%). Because each community meals program served identical meals to all of its diners on a given day (ie, did not allow diners choices), the amount of sodium served per diner was equivalent to the mean sodium content of meals served.

The recipes of 6 (4.1%) of the community meals programs’ 148 menu items were modified to reduce sodium content. For example, one community meals program replaced canned corn with frozen corn, which reduced the sodium content of the corn from 320 mg per serving (1/2 cup) at baseline to 0 mg per serving at follow-up. Two (1.4%) menu items were modified through ingredient or product substitution to reduce sodium content. For example, one community meals program stopped purchasing ranch salad dressing and began making honey mustard dressing on site. This substitution reduced the sodium content of dressing from 260 mg per serving (2 tablespoons) at baseline to 15 mg per serving at follow-up.

## Implications for Public Health

The northwest Arkansas SRCP project intervention yielded reductions in the amount of sodium served per diner during the evaluation period, reducing the amount sodium served to thousands of diners across the year in local schools and community meals programs. These results highlight the potential effectiveness of sodium reduction interventions focused on food service guidelines, procurement practices, food preparation practices, and environmental strategies for schools and community meals programs.

Overall, the evaluation findings address each SRCP evaluation question. Collectively, the findings establish evidence of the effectiveness of SRCP interventions in reducing the amount of sodium served in schools and community meals, contributing to the evidence base established by evaluations of SRCP activities in other venues in other communities (32–34). A key characteristic underlying the effectiveness of SRCP interventions is likely their comprehensive approach to sodium reduction, implicating food service guidelines, procurement practices, food preparation practices, and environmental strategies.

However, the comprehensive nature of the intervention is also a potential weakness. For example, intervention implementation was time and staff-intensive, relying on technical expertise of registered dietitians and experienced implementation staff, as well as intensive collaboration with venue personnel. Results in one community for one venue may not be easy to replicate in a similar venue in a different community. In addition, the comprehensive nature of the intervention makes it difficult to determine whether certain components of the intervention were more effective or less effective than others.

An additional limitation of the study is the evaluation approach itself. The intensive nature of negotiating access to data, data collection, and data processing for each participating site precluded the use of control groups. The lack of control groups leaves open the possibility of a general trend toward sodium reduction across schools and community meals, whether they had participated in the intervention or not. Similarly, the evaluation focused on measures of food served rather than food consumed. Although our study was designed to evaluate changes in the amount of sodium served to diners, it does not provide precise measures of the amount of sodium consumed or the ratio of sodium served to sodium consumed, which could have varied in unexpected ways from baseline to follow-up. Likewise, the decision to rely on nutrient databases rather than laboratory analysis of foods served raises the possibility of error based on discrepancies between the database entries and what was actually served to diners. However, a strength of the use of nutrient databases was that evaluation results included every food item served, which would have been prohibitively time-consuming and expensive had we used laboratory analysis.

Limitations notwithstanding, our evaluation study sampled the entire population of diners and meals served in participating schools and community meals programs and showed an 11.1% to 16.6% reduction in sodium per diner per school lunch or community meal. These percentages are consistent with health impact assessment models that predict sizeable health benefits of reduced sodium intake (15). These levels of sodium reduction suggest that SRCP’s policy, systems, and environmental approaches to intervention have promise in schools and community meals programs, including those that serve racial/ethnic minority, low-income, and food-insecure populations at risk for hypertension.

Although these initial results are promising, evaluation of Years 2 to 5 of the project will demonstrate whether reduction in daily sodium intake is sustained, is improved, or erodes. In Years 2 to 5, UAMS will implement additional intervention components in both venues to promote even greater sodium reduction. For example, UAMS will implement product placement interventions in school cafeterias, moving unflavored (ie, lower-sodium) milk to the front of beverage coolers. Likewise, UAMS will offer training in knife skills and fruit and vegetable preparation to food service staff in both venues to increase feasibility of incorporating fresh, low-sodium ingredients in meals. In addition, UAMS will seek partnership opportunities to implement sodium reduction interventions with additional school districts and community meals programs and has begun work in a third venue, early childhood nutrition programs operated by the Arkansas Department of Human Services.
